# Current Challenges in Diagnosis and Treatment of Cardiovascular Disease

**DOI:** 10.3390/jpm14080786

**Published:** 2024-07-25

**Authors:** George Samanidis

**Affiliations:** Department of Adult Cardiac Surgery, Onassis Cardiac Surgery Center, 17674 Athens, Greece; gsamanidis@yahoo.gr; Tel.: +30-2109493832

Cardiovascular disease is a leading the cause of death worldwide among the various cardiac pathologies that directly or indirectly affect the quality of life of patients. A current challenge in the management of cardiovascular disease is combining known clinical practice with updated diagnostic methods and treatment. Artificial intelligence may offer an alternative diagnostic approach for treating patients with cardiac disease, but alone and without clinician support, it may have limited application.

Cardiovascular disease is a leading cause of death worldwide [1]. A wide spectrum of cardiac pathologies directly or indirectly affect the quality of life of patients. Coronary artery disease, heart valve disease, thoracic aorta disease, heart rhythm disease and cardio-oncology are the most common heart pathologies. The diagnostic approaches used for treating these syndromes have changed in the last few years. Noninvasive diagnostics methods for heart disease diagnosis, such as coronary artery computed tomography (CT), cardiac CT, cardiac magnetic resonance imaging (MRI) and cardiac positron emission tomography (PET), are prominent strategies present in the armentaria of cardiologists, radiologists and cardiac surgeons [2,3,4]. Moreover, less invasive intervention techniques [transcather aortic valve implantation (TAVI), transcather mitral valve implantation (TMVI), Mitral-clip, endovascular thoracic and aortic arch branched stenting] allow patients to receive the most up-to-date treatment with acceptable short-, mid- and long-term results [5,6,7,8]. Unlike in the past, nowadays, inoperable patients or patients with a high or prohibitive perioperative cardiac surgery risk can be treated with modern transcather techniques. Improving the quality of mechanical circulatory support devices contributes to improved quality of life in patients with acute or chronic heart failure [9,10]. The number of patients with congenital heart disease who previously underwent heart surgery and adult patients with congenital heart disease will increase in the next few years. An accurate diagnostic approach in patients with acquired and congenital heart disease will be needed to ensure the best medical or intervention treatment. Also, the prevention of coronary artery disease and identification of patients with silent ischemic heart disease may reduce the number of patients admitted in hospital with acute symptoms and avoid ischemic heart complications. In future, older patients will constitute the majority of patients with heart disease in need of cardiology or cardiac surgery interventions. The comorbidities of these patients will be crucial point for treatment. Postoperative psychological dysfunction is identified as a risk factor for worse outcomes in patients who underwent major cardiac surgery in [11]. The early identification and treatment of these patients may improve perioperative and long-term results.

Nowadays, the primary treatment approaches used in interventional cardiology and cardiac surgery are transcather intervention in heart valve disease and either minimally invasive cardiac surgery or a hybrid approach in coronary artery disease and structural heart disease (aortic, mitral and tricuspid valves) [5,12,13]. Although transcatheter aortic valve implantation (TAVI) was introduced over 15 years ago, its long-term results regarding quality of life are still debated through comparison to the results of surgical aortic valve replacement. On the other hand, patients facing high or prohibitive risk in open cardiac surgery are suitable for TAVI with acceptable mid-term results. Severe mitral valve regurgitation in patients at high risk during an operation requires them to have alternative options for treatment by transcather mitral valve implantation (TMVI) [14]. Moreover, patients with severe mitral valve annulus calcification (MAC) who are not candidates for operation constitute a population suitable for TMVI. Many prosthetic mitral valves for TMVI have been developed. The short-term results after TMVI are encouraging, but the mid- and long-term results are unknown.

Minimizing surgical stress enables the fast recovery of patients who underwent major heart surgery [15]. Minimal invasive cardiac surgery (MICS) for aortic and mitral correction or replacement by a thoracoscopic approach are alternative surgical approaches. Making a small skin incision and avoiding median sternotomy reduces surgical stress and contributes to the early mobilization of patients after operation. These advantages prevent early postoperative complications such as lung atelectasis and pneumonia. Furthermore, an MICS approach reduces postoperative pain, which improves pulmonary function postoperatively. Short-, mid- and long-term results are similar with an open-sternum approach.

Coronary artery bypass grafting (CABG) remains the most common cardiac surgery procedure. On the other hand, a hybrid approach for treating severe coronary artery disease may offer better long-term results compared with percutaneous coronary intervention or optimal medical treatment in selective patients with coronary artery disease. Although the number of patients treated with a hybrid revascularization approach worldwide is small, the outcomes in these patients are promising [12]. More studies are needed to confirm these results and enable this method’s acceptance as a revascularization strategy in patients with severe coronary artery disease.

Diagnostic approaches used for the treatment of patients with progressive heart failure (HF) face many challenges [16,17,18]. Patients with preserved or non-preserved heart failure constitute large populations who need special therapy approaches. The use of alternative drug administration to achieve the optimum drug affect is well described by Sezai et al. [16]. Although the number of patients in this study is small and it has many weaknesses, the use of a transdermal patch for drug administration may be more effective than oral administration in patients with arterial hypertension and heart failure. Limited data are available about mental disorders and outcomes in patients undergoing heart transplantation or supported by mechanical circulatory support. Alyaydin et al., in their study, present the incidence of depression and anxiety in heart transplant patients [17]. The authors conclude that these patients have worse outcomes compared with patients without mental disorders. The study shows that chronic diseases including chronic heart disease affect direct quality of life and outcomes. A priority for the prevention of heart disease is the identification of patients in the early stage of progressive of heart disease. Chang et al. show that proteinuria may be a possible predictor of heart failure [18].

The many innovative diagnostic and treatment approaches for heart disease could also lead to a new era in medicine and cardiovascular disease. The use of up-to-date prosthetic heart valve devices for the transcatheter treatment of heart valve disease improves patient outcomes. Minimally invasive cardiac surgery minimizes surgical stress, and its use is a growing trend in cardiac surgery. On the other hand, the use of artificial intelligence in diagnostic methods (such as CT, MRI and transthoracic and transesophageal echocardiography) and diagnostic algorithms for heart disease diagnosis in routine clinical practice is currently a hot topic in cardiology and cardiac surgery [19,20,21,22,23]. Using artificial intelligence for the early identification of patients with severe heart disease and prediction of outcomes in these patients may improve such outcomes. Although artificial intelligence is hot topic in current medical practice, the clinical examination of patients, identification of crucial symptoms in patients, selection of laboratory tests and appropriate diagnostic examination remain the most significant elements of good medical practice. The application of artificial intelligence may offer the best diagnostic approach for patients with severe heart disease.

An interesting review about the possible cause of cerebral aneurysm is presented by Chojdak-Łukasiewicz et al. [24]. The authors, in their study, list cases of patients with coexisting he cardiac myxoma and cerebral aneurysm. The proposed cause of cerebral aneurysm formation is cerebral artery embolism or vascular inflammation due interleukin secretion from cardiac myxoma. No guidelines exist regarding the management and treatment of these patients. On the other hand, complete surgical excision remains the treatment of choice of patients with cardiac myxoma.

Yeh et al. compare woman of reproductive age with and without primary dysmenorrhea [25]. The authors present a large study population in comparative groups. In this study, the authors evaluate primary dysmenorrhea as a possible risk factor for late stroke. They conclude that dysmenorrhea is risk factor for stroke (ischemic and hemorrhagic) and affects patients’ quality of life.

In conclusion, the main challenge in the management of cardiovascular disease is the combination of known clinical practice with up-to-date diagnostic methods and treatments. Artificial intelligence may offer an alternative diagnostic approach for treating patients with cardiac disease, but alone and without clinician support, it may have limited application.
